# Fluoride release and uptake abilities of different fissure sealants

**DOI:** 10.4317/jced.52775

**Published:** 2016-07-01

**Authors:** Claudio Poggio, Gianluigi Andenna, Matteo Ceci, Riccardo Beltrami, Marco Colombo, Lucia Cucca

**Affiliations:** 1 MD, DDS. Department of Clinical-Surgical, Diagnostic and Pediatric Sciences - Section of Dentistry, University of Pavia, Italy; 2 DMD, PhD. Department of Clinical-Surgical, Diagnostic and Pediatric Sciences - Section of Dentistry, University of Pavia, Italy; 3 DMD, PhD. Department of Brain and Behavioural Sciences - Section of Statistics, University of Pavia, Italy; 4 MD, DDS. Department of Chemistry, University of Pavia, Italy

## Abstract

**Background:**

The long-term capability of resin sealants and glass ionomer cements to release fluoride is associated to a reduction in pit and fissure caries. The regular use of fluoride varnishes/toothpastes can result in the absorption of fluoride into the sealant. The objective of the present study was to assess the fluoride release/uptake capacities of different fissure sealants.

**Material and Methods:**

Three different fissure sealants (Fuji Triage/GC, Fissurit FX/Voco and Grandio Seal/Voco) were examined. Ten discs of each material were prepared. Each disc was incubated with distilled water and then the solution analyzed for diluted for fluoride concentration, using a combination of fluoride electrode (OrionGP 1 S/N 13824, Orion Research Inc, Boston, MA, USA) connected to an expandable ion analyzer (Orion 720A, Orion Research Inc, Boston, MA, USA). Standard curves between 1 and 100 ppm F- were used to calibrate the electrode. Cumulative fluoride release was measured on days 1, 2, 3, 5, 7, 21, 35 and 49, then two different fluoride varnishes/pastes (Profluorid Varnish/Voco, MI Paste Plus/GC), were applied to the sealants tested, and fluoride release (after reuptake) was measured on days 56, 70 and 84.

**Results:**

Kruskal Wallis test confirmed significant differences in fluoride release between Fuji Triage/GC and Fissurit FX/Voco and Grandio Seal/Voco from day 1 (*P* < 0.001). The application of fluoride varnish Profluorid Varnish enhanced the fluoride release for all sealants (*P* < 0.05). MI Paste Plus enhanced the fluoride release for all sealants except for Fuji Triage/GC (*P* > 0.05).

**Conclusions:**

The GIC-based sealant (Fuji Triage/GC) released significantly more fluoride than the resin sealants tested. The exposure to the fluoridated varnish (Profluorid Varnish) significantly recharged the sealants tested more than the CPP-ACPF toothpaste (MI Paste Plus).

** Key words:**Fissure sealants, fluoride release, fluoride uptake, glass ionomer cements.

## Introduction

Since 1960s, sealing the pits and fissures of molars and premolars is considered a highly effective method for the prevention of dental caries (1). The two predominant types of dental sealant nowadays are resin-based and glass ionomer cement (GIC) sealants (2).

To increase the caries-preventing effect, fluoride-releasing sealants were introduced in the 1970s (3). Current commercial fluoride-releasing sealants contain either a soluble fluoride salt such as sodium fluoride (NaF) or fluoride-releasing glass filler or both. Various *in vitro* studies reported that these materials can release fluoride and inhibit demineralization of the adjacent tooth structure (4). The long-term capability of resin sealants and glass ionomer cements to release fluoride to the sealed enamel and the adjacent unsealed pit and fissure is in fact associated to a reduction in pit and fissure caries experience for children (5).

As concerns Glass-Ionomer Cements (GICs), the fluoride release of these materials into the oral cavity is documented. Several *in vitro* and in situ studies have also shown that release of fluoride either in saliva or dental plaque from glass ionomer materials is thought to protect the tooth against dental caries (6). This benefit of fluoride release and subsequent adsorption is found not only in enamel immediately adjacent to glass ionomer restorations, but also in areas up to 3 mm away from the restoration margins and may even protect the entire tooth (7).

The concept that glass ionomer and resin-based sealants can act as rechargeable fluoride release devices has been proposed (8). Studies reported that the regular use of fluoride toothpastes can result in the absorption of fluoride into the glass ionomer and that this fluoride can subsequently be released into the adjacent tooth structure (9). *In vitro* and in situ investigations found that glass-ionomer and resin-modified glass-ionomer restorations in direct contact to adjacent teeth prevent demineralization compared to non-fluoridated amalgam and composite (10). However, additional application of topical fluoride (fluoridated dentifrice) significantly increased remineralization and decreased demineralization, respectively, in all groups independent from the material (11). This was also confirmed in another in situ investigation, where glass-ionomers showed some slight caries-preventive effects compared to a fluoridated and a non-fluoridated composite when subjects performed oral hygiene without topical fluorides. The regular application of fluoridated toothpaste was, however, effective in prevention of secondary caries in all experimental groups (12).

The objective of the present study was to assess the fluoride release/uptake capacities of different fissure sealants. The null hypothesis of the study was that there is no significant difference in fluoride release and uptake capacities between the fissure sealants tested.

## Material and Methods

-Specimens’ preparation 

Three different fissure sealants (Fuji Triage/GC, Fissurit FX/Voco and Grandio Seal/Voco) were examined ( Table 1). Cylindrical test specimens were made by placing each material into a metallic split mold, with inside dimensions of 8 mm (diameter) by 2 mm (height). The surface area of the cylindrical specimens was approximately 1.51 cm2.

**Table 1:**
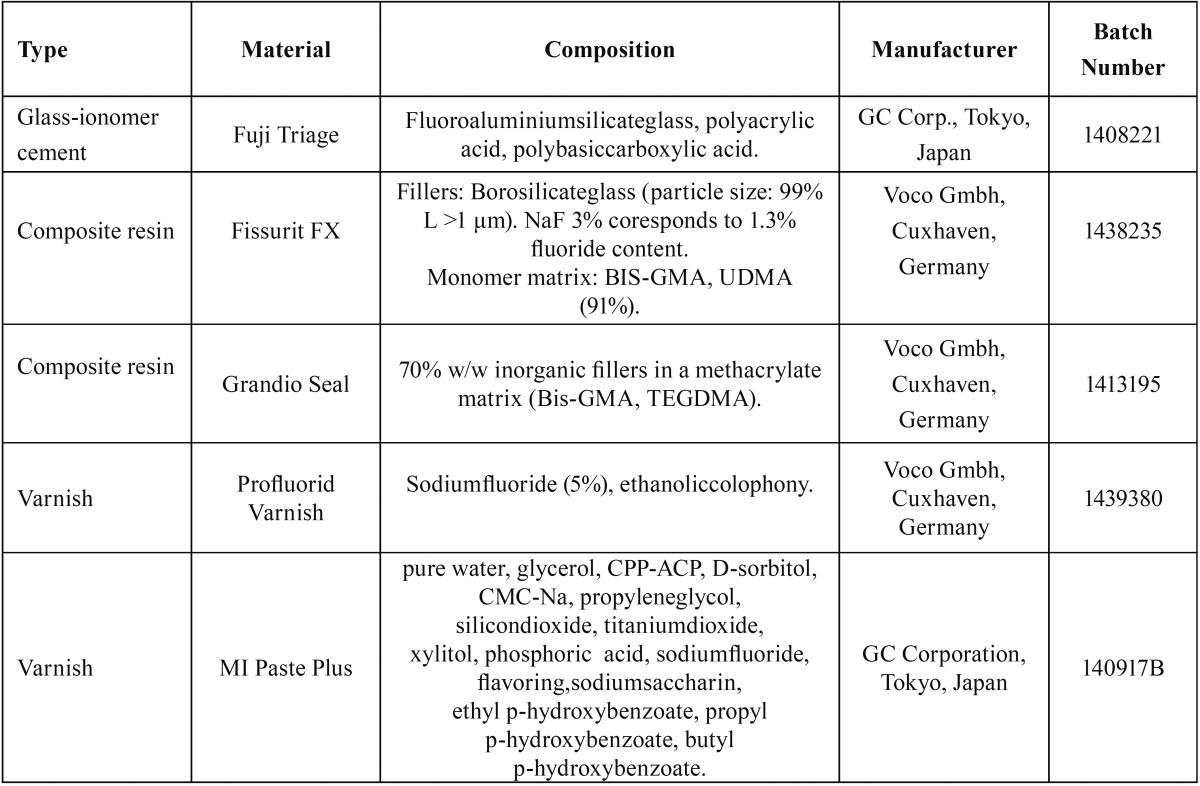
Fissure sealants and varnishes tested in the study.

The cements were prepared according to the manufacturer’s instructions and divided into 3 test groups. Each group consisted of 10 specimens. The materials were allowed to harden for 10 min during setting at room temperature. The surface of all materials was covered with a glass plate. No protective varnish was applied. The surfaces were not finished or polished.

-Fluoride release

After hardening, each specimen was removed from the mold, moved into polypropylene tube containing 3 mL deionized water and incubated at 37 °C for different days. Each sample was then rinsed with 1 mL of deionized water. The rinsed water was collected in the same tube and the specimen was transferred into a new tube with 3 mL deionized water. Each water sample was diluted V/V with buffer solution TISAB III (Orion Research Inc, Boston, MA, USA) and measured for fluoride concentration, in ppm, under stirring condition, using a combination of fluoride electrode (Orion GP 1 S/N 13824, Orion Research, Inc, Boston, MA, USA) connected to an expandable ion analyzer (Orion 720A, Orion Research Inc, Boston, MA, USA). Standard curves between 1 and 100 ppm F ¯ were used to calibrate the electrode. Cumulative fluoride release was measured at 1, 2, 3, 5, 7, 21, 35, and 49 days of specimen incubation.

-Release after fluoride exposure (uptake)

After 7 weeks the specimens were individually rinsed with 1 mL deionized water and allowed to air dry for 1 min. Two different fluoride varnishes/pastes ( Table 1, Table 2) were applied to the sealants tested. Each specimen was then rinsed three times with 3 mL deionized water, air dried for 1 min and placed in a tube containing 3 mL of deionized water, at 37 °C. Fluoride measurements were carried out at 56, 70 and 84 days.

**Table 2:**
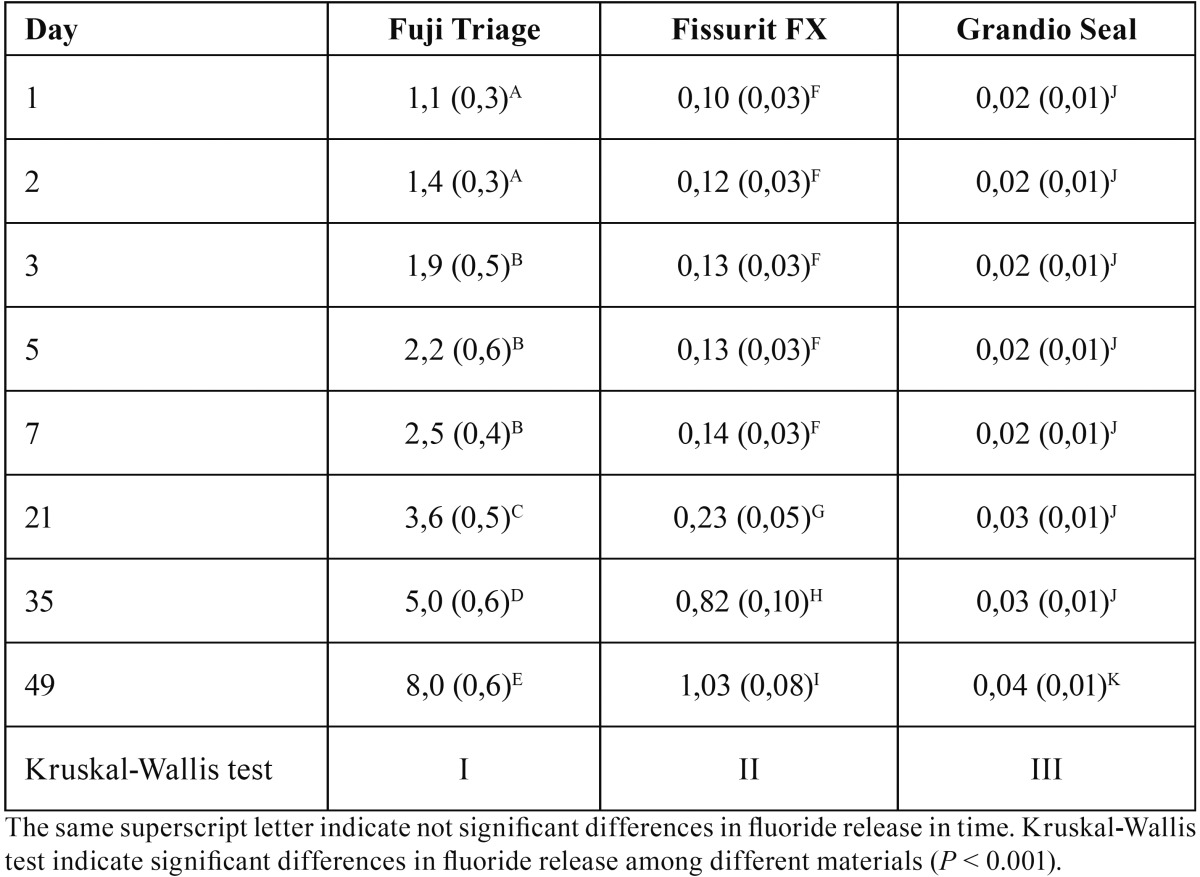
Medians (and standard deviation) of cumulative fluoride release values for each sealant in time (ppm F-).

-Statistical analysis

Statistical analysis was performed with Stata 12.0 Software (Stata Corp., College Station, TX). The Mead’s resource equation was used to determine the sample size for each experimental group. Descriptive statistics, including the mean, standard deviation, median, minimum and maximum values, were calculated for each of the 3 groups. Shapiro Wilk test was applied to test the normality of the distributions (*P* < 0.05). Kruskal Wallis not parametrical analysis of variance was applied to determine whether significant differences existed in fluoride release among the groups (*P* < 0.001). Wilcoxon test for paired data was applied to assess release after fluoride exposure (uptake) (*P* < 0.05).

## Results

**Figure 1:**
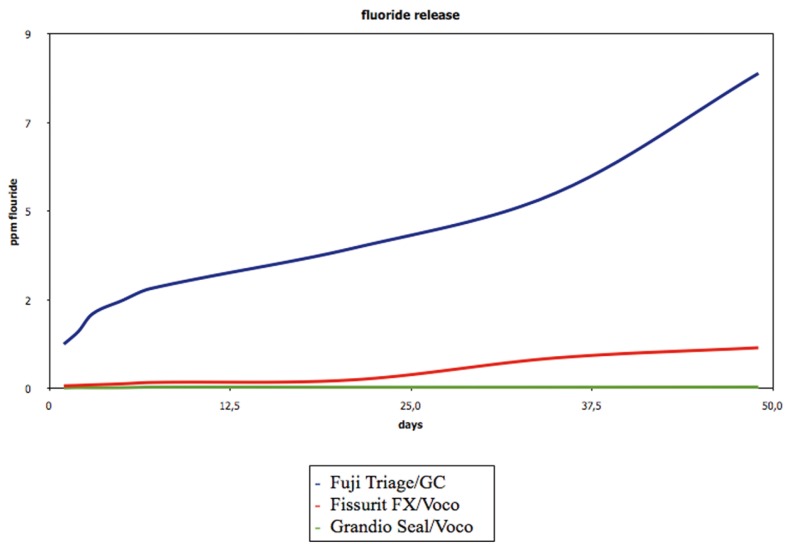
Cumulative fluoride release (in ppm) of three different sealants over time.

Descriptive statistics for cumulative fluoride release are presented in  Table 2 and  Table 3. Data are not normally distributed as confirmed by Shapiro Wilk test (*P* < 0.05). Kruskal Wallis test confirmed significant differences in fluoride release between Fuji Triage/GC and Fissurit FX/Voco and Grandio Seal/Voco from day 1 (*P* < 0.001). Significant differences emerged between Fissurit FX/Voco and Grandio Seal/Voco after 21 days (*P* < 0.05); Fissurit FX/Voco showed significantly higher fluoride release than Grandio Seal/Voco till day 49. Grandio Seal/Voco did not show any significant difference between day 1 and day 49 (*P* > 0.05) (Fig. 1). The application of fluoride varnish Profluorid Varnish enhanced the fluoride release for all sealants (*P* < 0.05). MI Paste Plus enhanced the fluoride release for all sealants except for Fuji Triage/GC (*P* > 0.05) but the quantity of fluoride release was significantly lower if compared with Profluorid Varnish (*P* < 0.001).

**Table 3:**
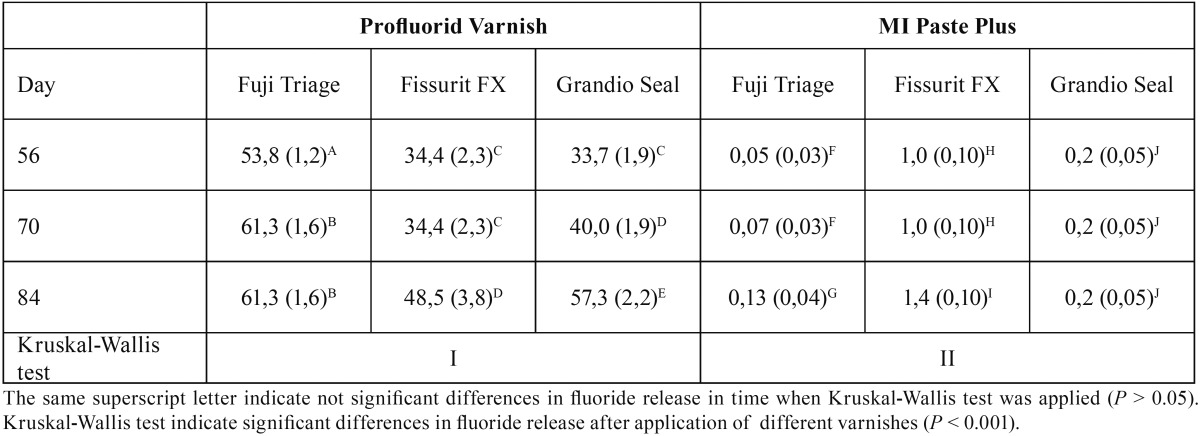
Medians (and standard deviation) of cumulative fluoride release values for each sealant after application of two different fluoride varnishes/pastes (ppm F-).

## Discussion

The null hypothesis of the study has been rejected. Statistical analysis confirmed significant differences in fluoride release between the three sealants tested.

In the present investigation, samples were stored in deionized water. Previous studies showed that fluoride release into artificial saliva is lower than into deionized water (13). The amount of fluoride release in deionized water could be different from the one found in the oral cavity, because saliva is a constantly changing medium, with respect of temperature, pH, protein content and many other factors (14). In the present study the presence of plaque or pellicle that may concentrate fluoride levels was not taken into account, according to other investigations (14,15).

All the fissure sealants evaluated in this study released measurable amounts of fluoride. This observation is consistent with the findings of many other authors (16). From day 1 to 49, Fuji Triage/GC showed significant higher fluoride release if compared to composite sealants. Furthermore, from day 21, Fissurit FX/Voco demonstrated significant higher fluoride release if compared to Grandio Seal/Voco. Grandio Seal/Voco reported the lowest fluoride release during the whole experimentation period. These results are explained by the different composition of the tested materials. Fuji Triage/GC consists of a Glass-Ionomer Cements (GICs) with fluoroaminosilicate glass and Fissurit FX/Voco is fluoride-releasing composite sealants with 3% NaF content. Contrariwise Grandio Seal/Voco is conventional resin sealants without addition of fluoride. Our findings are in agreement with recent studies: glass ionomer based sealants released significantly more fluoride than resin modified glass ionomer or resin composite sealants (17). In GlCs, there is an acid base reaction resulting in the leaching of Ca2+, Al3+ and F- ions to form a polysalt matrix. This may be responsible for the short term elution process. In composites, there is no acid-base reaction; the only source of fluoride would come from glass filler particles, resulting in a slow diffusive release (18).

Patterns of fluoride released from Fuji Triage/GC in deionized water showed higher release during the first week, with the most rapid release occurring in the first days, followed by a considerably less but continuous fluoride release for the next weeks. This is in agreement with previous studies (19). The higher fluoride release from glass ionomer cements during the first days is named “burst effect” by different authors (14). The reason of the rapid decrease of fluoride release during subsequent weeks is likely due to the initial burst of fluoride released from the glass particles as they dissolve in the polyalkeonate acid during the setting reaction. The later slow release occurs as the glass dissolves in the acidified water of the hydro gel matrix (20).

The ability of a restorative material to act as a fluoride reservoir is mainly dependent on the type and permeability of filling material, on the frequency of fluoride exposure and on the kind and concentration of the fluoridating agent (21). Glass-ionomers are mostly found to have significantly better capability to act as a fluoride reservoir than composite resin-based materials (22). This fact can be explained by the loosely bound water and the solutes in the porosities in the glass-ionomer, which may be exchanged with an external medium by passive diffusion. The absorption and re-release of fluoride might be determined by the permeability of the material. Thus, a completely permeable substance could absorb the ions deep into its bulk, while a relatively impermeable material can only absorb fluoride into the immediate subsurface (23). Therefore, the slight increase of fluoride release from re-sin-based restoratives after exposure to exogenous fluoride is discussed to be most probably because of surface-retained fluoride (24). In general, materials with higher initial fluoride release have higher recharge capability (23).

Profluorid Varnish is a colophony-based varnish containing 5% sodium fluoride (22,600 ppm fluoride). The fluoride ion, together with the calcium ions, causes a precipitation of calcium fluoride. MI Paste Plus contains 900 ppm fluoride and casein phosphopeptide-amorphous calcium phosphate (CPP-ACPF). Recent studies have investigated the remineralization potential of casein phosphopeptide-amorphous calcium phosphate combined with fluoride and have found a synergistic effect (25).

In the present study, after the exposure to fluoridated varnish/paste, fluoride release increased significantly. This is in agreement with previous studies that evaluated the effects of refluoridation of different sealants on fluoride release (14,26). However, the application of Profluorid Varnish enhanced the fluoride release for all sealants. Contrariwise MI Paste Plus enhanced the fluoride release for all sealants except for Fuji Triage/GC and the quantity of fluoride release was significantly lower if compared with Profluorid Varnish. Although no study has evaluated the use of a CPP-ACPF toothpaste to recharge fissure sealants; our results are in agreement with a recent study on the recharge abilities of different fissure sealants with various fluoride preventive treat-ments (varnishes or toothpastes). In this study, the use of fluoride varnish significantly recharged the sealants more than highly fluoridated toothpaste (27).

## Conclusions

Within the limitations of this *in vitro* study, the glass ionomer based sealant (Fuji Triage) released significantly more fluoride than the other tested fissure sealants (resin sealants) during the whole experimentation period. After the exposure to fluoridated varnish/paste, fluoride release increased significantly. Furthermore, the preventive treatment with fluoride varnishes (Profluorid Varnishes) significantly recharged all the sealants tested more than fluoridated CPP-ACP toothpaste (MI Paste Plus).
